# Fibroblasts as Immunological Sentinels in Cutaneous Inflammation: A Review

**DOI:** 10.3390/jcm15020556

**Published:** 2026-01-09

**Authors:** Taihao Quan

**Affiliations:** Department of Dermatology, University of Michigan Medical School, Ann Arbor, MI 48109, USA; thquan@umich.edu; Tel.: +1-(734)-615-2403; Fax: +1-(734)-647-0076

**Keywords:** fibroblasts, inflammatory mediators, inflammation, skin

## Abstract

Fibroblasts, traditionally viewed primarily as structural cells responsible for extracellular matrix production and tissue architecture, have emerged as important immunomodulatory players in inflammation. These cells actively participate in inflammatory processes through multiple mechanisms: recognizing and responding to inflammatory stimuli, producing diverse inflammatory mediators, and engaging in complex interactions with various immune cells. This review explores the multifaceted immunomodulatory functions of fibroblasts, including their capacity to sense inflammatory signals, secrete inflammatory mediators, modulate immune cell behavior, and establish a pro-inflammatory microenvironment. Understanding the dynamic role of fibroblasts in inflammatory processes provides insights into inflammatory pathology and may inform the development of novel therapeutic strategies targeting fibroblast-mediated immune modulation.

## 1. Introduction

The skin serves as the body’s largest organ and primary barrier against environmental insults, pathogens, and physical trauma. Fibroblasts, the predominant stromal cells within the dermis, have long been recognized for their essential structural functions, particularly in synthesizing and organizing the extracellular matrix (ECM) that provides structural and mechanical support to the skin [1,2].

The traditional view of fibroblasts as passive structural cells has undergone a paradigm shift in recent years. Once considered passive bystanders, fibroblasts are now recognized as active participants in inflammation through their ability to respond to inflammatory signals, produce inflammatory mediators, recruit immune cells, and modulate the immune microenvironment [3,4,5]. These immunomodulatory functions establish fibroblasts as important regulators of inflammation, shaping the initiation, progression, and resolution of inflammatory diseases.

Skin inflammation encompasses a broad spectrum of acute and chronic conditions, including psoriasis, atopic dermatitis, contact dermatitis, and fibrotic disorders. While these diseases have distinct etiologies and clinical manifestations, they share common pathogenic mechanisms involving dysregulated immune responses and aberrant tissue remodeling. Emerging research demonstrates that immunomodulatory fibroblasts play a significant role in these pathological processes by recognizing and responding to inflammatory stimuli, producing inflammatory mediators, and interacting with immune cells [4,6,7,8].

This review summarizes current knowledge on the immunomodulatory roles of fibroblasts in inflammation. It details fibroblasts’ capacity to recognize and respond to inflammatory stimuli, the mediators they produce, and their interactions with various immune cell populations. Finally, it discusses therapeutic implications and future research directions that may translate our understanding of fibroblast biology into novel treatment strategies for inflammatory skin conditions.

## 2. Fibroblasts as Sentinels: Sensing Inflammatory Stimuli

The primary role of fibroblasts is to synthesize ECM components such as collagens, elastin, fibronectin, and laminins, which contribute to connective tissue formation and maintain tissue structural integrity.

In recent years, however, the conventional perception of fibroblasts as merely passive structural cells has shifted, with growing evidence indicating that these cells play a more active role in immune surveillance and inflammation than previously appreciated [3,9]. Single-cell RNA sequencing and lineage tracing studies have revealed greater complexity within fibroblast populations, identifying functionally distinct subsets with specialized roles [10]. Dermal fibroblasts, in particular, exhibit marked transcriptional and functional heterogeneity, with distinct subsets actively participating in both innate and adaptive immune defense [6,7]. These immunomodulatory fibroblast populations exhibit certain transcriptional profiles and functional characteristics [7,11]. These cells are predominantly expanded and functionally activated within the context of diseases. While some marker genes and regulatory pathways are shared between universal dermal fibroblast subsets and those observed in disease states, the immunomodulatory phenotype is significantly more pronounced, or perhaps exclusive, to the disease environment.

The immunomodulatory fibroblasts can function as primary drivers of pathology through their capacity to initiate inflammatory responses and produce inflammatory mediators that orchestrate immune cell recruitment. Genetic manipulation studies demonstrate that fibroblast dysfunction alone is sufficient to trigger inflammatory skin disease. Specifically, targeted deletion of the *Ikkb* gene (encoding IKKβ) in fibroblasts resulted in spontaneous development of an atopic dermatitis-like phenotype characterized by eosinophilic infiltration and type 2 immune responses [12]. Mechanistically, this occurred through aberrant fibroblast expression of CCL11, which initiated eosinophilic and Th2 inflammation. Additional genetic studies further establish fibroblasts as primary drivers of pathology. Targeted ablation of specific fibroblast subsets, including CD26+ fibroblasts, Adam12+ myofibroblast progenitors, and FAP+ fibroblasts, using diphtheria toxin-based strategies effectively suppressed both fibrosis and inflammation [13]. Moreover, in mouse models of inflammatory arthritis, selective depletion of FAP-expressing stromal cells completely prevented disease progression [14]. Collectively, this evidence demonstrates that fibroblasts function as critical instigators of pathology through two principal mechanisms: their capacity to initiate inflammatory cascades when dysregulated, and their production of inflammatory mediators that orchestrate immune cell recruitment and perpetuate tissue damage.

The immunomodulatory fibroblasts can detect danger signals, secrete inflammatory mediators, attract immune cells, and influence immune responses. The fibroblasts express diverse pattern recognition receptors (PRRs) that enable detection of pathogen-associated molecular patterns (PAMPs) and damage-associated molecular patterns (DAMPs) [2,15]. These PRRs include toll-like receptors (TLRs), NOD-like receptors (NLRs), and RIG-I-like receptors (RLRs), which collectively allow fibroblasts to discriminate between various microbial components and endogenous danger signals. Upon PRR engagement, fibroblasts undergo rapid transcriptional reprogramming that orchestrates the production of inflammatory cytokines, chemokines, and antimicrobial peptides, thereby initiating and amplifying immune responses within the tissues.

### 2.1. Pattern Recognition Receptors in Fibroblasts

**Figure 1:**
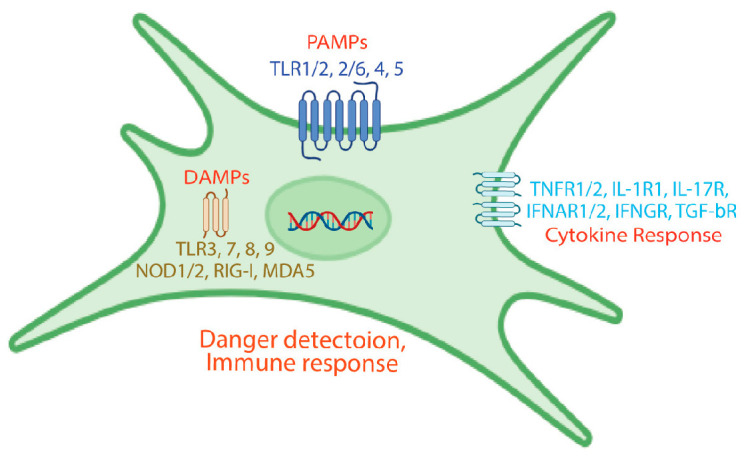
Fibroblasts express a diverse array of pattern recognition receptors (PRRs), allowing them to detect pathogen-associated molecular patterns (PAMPs) and damage-associated molecular patterns (DAMPs). Additionally, fibroblasts possess numerous cytokine receptors, enabling them to sense and respond to inflammatory cues from immune cells and other tissue residents.

Fibroblasts express a diverse and sophisticated repertoire of pattern recognition receptors (PRRs) that enable them to function as sentinel cells capable of detecting and responding to both pathogen-associated molecular patterns (PAMPs) and damage-associated molecular patterns (DAMPs) (Figure 1) [2,6,16,17]. This sensing capacity positions fibroblasts as sentinel cells capable of initiating inflammatory responses when tissue integrity is threatened. Toll-like receptors (TLRs) represent one of the most important PRR families expressed by fibroblasts [17,18,19,20,21,22,23]. These receptors recognize distinct molecular patterns: TLR2 detects bacterial lipopeptides and peptidoglycan; TLR3 recognizes double-stranded RNA; TLR4 responds to lipopolysaccharide (LPS) from Gram-negative bacteria; TLR5 detects flagellin; and TLR9 recognizes unmethylated CpG DNA motifs [17,24]. When fibroblasts encounter TLR ligands, they activate downstream signaling cascades involving adaptor proteins such as MyD88 and TRIF, leading to activation of transcription factors including NF-κB and interferon regulatory factors (IRFs) [25]. This signaling culminates in the production of pro-inflammatory cytokines, chemokines, and type I interferons. For example, stimulation of fibroblasts with LPS through TLR4 induces production of IL-6, IL-8, and monocyte chemoattractant protein-1 (MCP-1/CCL2), contributing to immune cell recruitment and amplification of inflammatory responses [17,22,26].

Beyond TLRs, fibroblasts express cytoplasmic PRRs including NLRs and RLRs [3,27]. NOD1 and NOD2 detect bacterial peptidoglycan fragments that enter the cytoplasm, activating NF-κB and MAPK signaling pathways. RIG-I and MDA5 recognize viral RNA species in the cytoplasm, triggering type I interferon responses. The expression and function of these cytoplasmic PRRs enable fibroblasts to respond to intracellular infections and cellular damage. Fibroblasts also respond to DAMPs released from damaged or stressed cells. These endogenous danger signals include high-mobility group box 1 (HMGB1), heat shock proteins (HSPs), extracellular ATP (eATP), monosodium urate (MSU) crystals, and extracellular matrix (ECM) breakdown products such as low molecular weight hyaluronan fragments and fibronectin extra domain A (EDA) fragments [28,29,30,31,32,33]. DAMPs can engage multiple receptors on fibroblasts, including TLRs, receptor for advanced glycation end products (RAGE), and purinergic receptors. DAMP recognition by fibroblasts contributes to sterile inflammation in conditions such as mechanical injury, UV exposure, and autoimmune diseases [34].

The expression and responsiveness of PRRs in fibroblasts can be modulated by the inflammatory milieu. Pro-inflammatory cytokines can regulate TLR expression, enhancing fibroblast sensitivity to PAMPs and DAMPs [35,36]. Conversely, certain anti-inflammatory mediators may downregulate PRR expression or signaling, serving as negative feedback mechanisms to limit excessive inflammation. The recognition of inflammatory stimuli through PRRs establishes fibroblasts as active participants in innate immune surveillance. By detecting and responding to danger signals, fibroblasts can initiate local inflammatory responses, alert immune cells to tissue threats, and coordinate the early phases of inflammation before adaptive immune responses are fully mobilized.

### 2.2. Cytokine Receptor Expression in Fibroblasts

In addition to PRRs, fibroblasts express numerous cytokine receptors that enable them to sense and respond to inflammatory signals from immune cells and other tissue residents (Figure 1) [21,37]. This extensive cytokine receptor repertoire positions fibroblasts as integration points for inflammatory signals, allowing them to modulate their functions based on the overall inflammatory context.

Fibroblasts express receptors for major pro-inflammatory cytokines, including IL-1 receptor (IL-1R), TNF receptors (TNFR1 and TNFR2), and IL-17 receptor (IL-17R) [6,7,11]. Activation of these receptors triggers intracellular signaling cascades that profoundly alter fibroblast behavior. IL-1β signaling through IL-1R activates NF-κB and MAPK pathways, inducing production of secondary inflammatory mediators [38,39]. IL-17A, often elevated in inflammatory skin diseases such as psoriasis, signals through IL-17R to induce fibroblast production of chemokines and antimicrobial peptides [40,41].

Interferon receptors are also prominently expressed on fibroblasts [42,43]. Type I interferon receptors (IFNAR1/IFNAR2) respond to IFN-α and IFN-β, which are produced during viral infections and autoimmune responses. Type II interferon receptor (IFNGR) recognizes IFN-γ, a key cytokine produced by T cells and natural killer cells. Interferon signaling activates JAK-STAT pathways in fibroblasts, leading to expression of interferon-stimulated genes (ISGs) that have antiviral, immunomodulatory, and growth-regulatory functions [44,45]. Chronic interferon signaling can contribute to fibroblast dysfunction in conditions such as systemic sclerosis and lupus erythematosus [46,47].

The expression of anti-inflammatory cytokine receptors on fibroblasts provides mechanisms for inflammation resolution. IL-10 receptors (IL-10R) enable fibroblasts to respond to this key anti-inflammatory cytokine, which can suppress fibroblast production of pro-inflammatory mediators and promote tissue repair [48]. Similarly, receptors for TGF-β and other regulatory cytokines can shift fibroblasts toward immunosuppressive or tissue-remodeling phenotypes.

Fibroblast cytokine receptor expression is dynamically regulated through transcriptional, post-transcriptional, and post-translational mechanisms, creating complex feedback circuits that modulate inflammatory responses [49,50]. Pro-inflammatory cytokines (TNF-α, IL-1β, IFN-γ) can upregulate or downregulate their cognate receptors and cross-regulate heterologous receptor expression, establishing both amplifying (positive feedback) and dampening (negative feedback) loops that determine the magnitude and duration of fibroblast activation [51,52]. For example, chronic exposure to certain cytokines may lead to receptor downregulation and desensitization, while other conditions may sensitize fibroblasts to specific signals. Through their extensive cytokine receptor repertoire, fibroblasts integrate multiple inflammatory signals and coordinate appropriate responses. This positions fibroblasts not merely as responders to inflammation but as computational centers that process diverse inputs and generate contextualized outputs that shape tissue-level inflammatory dynamics.

## 3. Fibroblast-Derived Inflammatory Mediators

Fibroblasts produce a diverse repertoire of inflammatory mediators, including cytokines, chemokines, growth factors, and lipid mediators, that collectively orchestrate immune cell recruitment, activation, and regulation.

### 3.1. Cytokine Production

Fibroblasts are prolific producers of cytokines, contributing substantially to the inflammatory milieu. Upon activation by inflammatory stimuli, fibroblasts secrete a diverse array of cytokines that can amplify inflammation, recruit immune cells, and influence the differentiation and function of other cell types (Table 1).

Interleukin-6 (IL-6) represents a major cytokine secreted by activated dermal fibroblasts and is among their most abundantly produced inflammatory mediators [21,53,54]. IL-6 is a pleiotropic cytokine with both pro-inflammatory and regulatory functions. Fibroblast-derived IL-6 promotes the acute phase response, enhances B cell antibody production, and influences T cell differentiation, particularly favoring Th17 cell development in the presence of TGF-β. In inflammatory skin diseases, elevated IL-6 levels contribute to systemic manifestations and local tissue pathology [55]. Stimuli including IL-1β, TNF-α, and TLR ligands potently induce IL-6 production by fibroblasts through NF-κB-dependent mechanisms.

Interleukin-8 (IL-8/CXCL8), while a CXC chemokine, also functions as a pro-inflammatory cytokine. Fibroblasts produce substantial amounts of IL-8 in response to inflammatory stimulation [41]. IL-8 serves as a potent chemoattractant and activator of neutrophils, and it also affects other cell types including T cells and keratinocytes. Fibroblast-derived IL-8 plays a critical role in neutrophil recruitment during acute inflammatory responses and sustains neutrophilic infiltration in chronic inflammatory conditions [56]. As a potent chemokine, IL-8 establishes chemotactic gradients that direct neutrophil migration to sites of tissue injury or infection, perpetuating the inflammatory cycle in diseases characterized by persistent neutrophil accumulation.

Fibroblasts produce IL-1 family members, including IL-1α and IL-1β, though typically at lower levels than typical immune cells [57,58]. However, fibroblast-derived IL-1 can contribute to autocrine and paracrine signaling loops that amplify inflammatory responses [59,60]. IL-1α, which can be released from damaged cells, is particularly important in initiating sterile inflammation [61]. Fibroblasts also produce IL-1 receptor antagonist (IL-1Ra), a natural inhibitor of IL-1 signaling, suggesting they participate in both promoting and regulating IL-1-mediated inflammation [62].

Fibroblasts produce members of the IL-10 family, including IL-10 itself, though this production is often context-dependent [21,63]. IL-10 is a key anti-inflammatory cytokine that suppresses production of pro-inflammatory mediators and promotes inflammation resolution. The capacity of fibroblasts to produce IL-10 suggests they can participate in negative feedback regulation of inflammation, though this function may be impaired in chronic inflammatory conditions.

Colony-stimulating factors (CSFs), including granulocyte-macrophage colony-stimulating factor (GM-CSF) and macrophage colony-stimulating factor (M-CSF), are produced by activated fibroblasts [21,64,65,66]. These factors influence the recruitment, differentiation, and activation of myeloid cells. GM-CSF produced by fibroblasts and other stromal cells enhances the inflammatory functions of macrophages and dendritic cells by promoting M1 polarization and pro-inflammatory cytokine production, while M-CSF supports macrophage survival, proliferation, and alternative M2 polarization toward tissue-resident, anti-inflammatory phenotypes [66].

While not a primary source, fibroblasts can produce TNF-α under certain conditions, particularly following strong inflammatory stimulation [14,67]. Fibroblast-derived TNF-α may contribute to local amplification of inflammatory signals and can act in autocrine fashion to further activate fibroblasts themselves. Fibroblasts produce members of the TNF superfamily beyond TNF-α, including BAFF (B cell activating factor) and APRIL (a proliferation-inducing ligand), which influence B cell function and survival [21,68,69,70].

The production of type I interferons (IFN-α/β) by fibroblasts occurs primarily in response to viral infections or intracellular nucleic acid sensing [9,21]. Fibroblast-derived type I interferons contribute to antiviral defenses but can also promote inflammation in autoimmune conditions. The dysregulation of the interferon pathway in fibroblasts has been implicated in diseases such as dermatomyositis and systemic lupus erythematosus.

**Table 1:** Cytokines secreted by fibroblasts.

Cytokine	Function	References
IL-1	proinflammatory/chemoattractant for T and B cells	[64,65]
Il-6	proinflammatory/chemoattractant for nuetrophils and T cells	[26,60,61]
IL-8	proinflammatory/chemotaxis	[48,63]
IL-10	anti-inflammatory/Inhibits production of TNF-α, IL-1, IL-6, IL-12	[26,71]
CSFS	stimulate production, differentiation, and function of blood cells	[72,73,74]
TNF-a	proinflammatory/activate T cells, macrophages, and granulocytes	[18,75]
BAFF	survival and maturation of B lymphocytes	[58,75,76]
APRIL	Enhances B cell maturation and differentiation	[58,75,77]
INF-a/β	Promotes adaptive immune responses	[9,26]

### 3.2. Chemokine Secretion

Chemokines, a family of small chemotactic cytokines, are among the most important mediators produced by fibroblasts in inflammatory contexts [3,4,21]. Through chemokine secretion, fibroblasts orchestrate the recruitment of specific immune cell populations to inflamed tissue, thereby shaping the composition and magnitude of inflammatory infiltrates. Fibroblasts produce numerous CC chemokines, which primarily attract mononuclear cells (Table 2).

CCL2 (MCP-1) is abundantly secreted by fibroblasts in response to inflammatory stimulation [78]. As a potent chemoattractant for monocytes and macrophages, fibroblast-derived CCL2 plays a critical role in recruiting these immune cells to inflamed tissues [71]. Elevated CCL2 expression has been documented across multiple inflammatory diseases, where fibroblast production of this chemokine contributes to the sustained macrophage infiltration that characterizes chronic inflammation [72,73].

CCL5 (RANTES) is another CC chemokine prominently produced by activated fibroblasts [3,9,21]. CCL5 attracts T cells, monocytes, and eosinophils, contributing to the mixed cellular infiltrates observed in many inflammatory skin conditions. CCL5 production by fibroblasts is induced by inflammatory cytokines, particularly IFN-γ and TNF-α, linking T cell activation to enhanced recruitment of additional immune cells.

CCL17 (TARC) and CCL22 (MDC) are chemokines that bind to CCR4 and preferentially attract Th2 cells and regulatory T cells. Fibroblast production of these chemokines has been implicated in atopic dermatitis and other Th2-dominated conditions [4,74]. The ability of fibroblasts to produce CCL17 and CCL22 suggests they can selectively recruit specific T cell subsets based on the inflammatory context.

CCL20 (MIP-3α) is particularly interesting because it attracts cells expressing CCR6, including Th17 cells, regulatory T cells, and immature dendritic cells [75]. Fibroblast production of CCL20 is strongly induced by IL-17 and IL-22, creating a positive feedback loop whereby Th17 cells induce fibroblasts to produce chemokines that recruit additional Th17 cells [4,7]. This mechanism has been implicated in psoriasis pathogenesis.

Among CXC chemokines, fibroblasts produce CXCL8 (IL-8) [76], CXCL1 [77], CXCL2 [79], and CXCL3 [80], which bind to CXCR1 and CXCR2 and primarily attract neutrophils [56,81,82]. The production of these chemokines enables fibroblasts to recruit neutrophils during acute inflammation or in chronic conditions characterized by neutrophilic infiltration. CXCL8 production by fibroblasts is potently induced by IL-1β, TNF-α, and bacterial products, positioning fibroblasts as key regulators of neutrophil recruitment in response to infection or tissue damage [14].

CXCL9 (MIG), CXCL10 (IP-10), and CXCL11 (I-TAC) are interferon-inducible CXC chemokines that bind to CXCR3 and attract activated T cells, particularly Th1 cells, and NK cells [9,21,81]. Fibroblast production of these chemokines is strongly induced by IFN-γ and, to a lesser extent, by type I interferons [21,81]. The CXCR3 ligand system plays important roles in T cell-mediated inflammatory responses, and fibroblast-derived CXCR3 ligands contribute to T cell recruitment in conditions such as contact dermatitis and cutaneous lupus.

CXCL12, which binds to CXCR4, is constitutively expressed by fibroblasts and plays roles in maintaining tissue architecture and stem cell niches [11,83,84,85,86]. CXCL12 primarily attracts cells expressing its main receptor, CXCR4, including T cells, monocytes, neutrophils, and B cell precursors. CXCL12, also known as fibroblast-specific protein (SDF1), is significantly elevated in dermal fibroblasts in psoriatic inflammatory skin lesions [11,87]. Inhibition of fibroblast-derived CXCL12 promotes wound regeneration and prevents fibrotic scarring by regulating immune responses [88]. CXCL12+ fibroblast subsets in mouse skin orchestrate neutrophil recruitment during *S. aureus* infection by sensing IL-17 and TNFα and releasing CXCR2 ligands and CXCL12 via NFKBIZ signaling [11]. Fibroblast-specific *Il17ra* deletion impaired neutrophil recruitment and increased infection, establishing CXCL12+ dermal fibroblasts as critical regulators of neutrophil-mediated host defense.

**Table 2:** Chemokines secreted by fibroblasts.

Chemokine	Other Names (Human)	Function (Typical Targets)	References
CCL2	MCP-1	monocyte, T cells, dendric cells	[79,80,81]
CCL5	RANTES	T cells, monocytes, eosinophils	[9,26]
CCL17	TARC	Th2 cells, Treg cells	[14,83]
CCL20	MIP-3a LARC	Th17 cells, T reg cells	[84]
CCL22	MDC	Th2 cells, Treg cells	[85]
CXCL1	GROa, MGSA	neutrophils	[87]
CXCL2	GROb	neutrophils	[88]
CXCL3	GROg	neutrophils	[89]
CXCL8	IL-8	neutrophils	[86]
CXCL9	MIG	Th1 cells, NK cells	[26,90]
CXCL10	IP-10	Th1 cells, NK cells	[26,90]
CXCL11	I-TAC	Th1 cells, NK cells	[9,26]
CXCL12	SDF-1	T cells, B cells, monocytes, neutrophils	[14,91,92,93]

### 3.3. The Role of TGF-β Signaling in Fibroblasts: Mechanical Priming and Myofibroblast Transition

Fibroblasts express receptors for TGF-β family members, which play dual roles in inflammation and fibrosis [89,90]. While TGF-β can dampen certain inflammatory responses [9,94,95], it also drives myofibroblast differentiation and pathological fibrosis [91,92,93], highlighting the complex role of this pathway in inflammatory skin diseases.

The interplay between TGF-β signaling and mechanical forces in fibroblast biology represents a critical regulatory axis in tissue repair and fibrosis [92,96]. Fibroblasts exist in a mechanically sensitive stromal microenvironment, where substrate stiffness and mechanical tension serve as crucial determinants of cellular responsiveness to TGF-β [97]. Specifically, mechanical priming, the exposure of fibroblasts to increased ECM stiffness or mechanical stress, significantly enhances their susceptibility to TGF-β-induced myofibroblast differentiation [98]. This process involves the upregulation of α-smooth muscle actin (α-SMA) and the formation of stress fibers, hallmark features of the contractile myofibroblast phenotype [99].

The mechanotransduction pathways including integrins, focal adhesions, and cytoskeletal tension act as biophysical sensors that modulate TGF-β receptor signaling [100]. Critically, mechanical forces can activate latent TGF-β through integrin-mediated mechanisms, creating a feed-forward loop where tissue stiffening promotes further TGF-β activation and fibroblast-to-myofibroblast transition [93,100]. This bidirectional relationship between mechanical and biochemical signaling helps explain the persistence of fibrosis even after initial injury resolution.

Furthermore, matrix stiffness alone, in the absence of soluble TGF-β, can induce proto-myofibroblast formation, while full myofibroblast differentiation requires both mechanical and TGF-β signals [92,101]. This concept has profound implications for understanding chronic inflammatory fibrotic diseases, where progressively stiffening tissue creates a permissive environment for sustained myofibroblast activity and excessive ECM deposition [102].

In the context of tissue damage and repair, these findings illuminate how the fibrotic cascade becomes self-perpetuating: initial injury triggers both inflammatory TGF-β release and alterations in ECM mechanics, which together drive myofibroblast differentiation [96,103,104]. These activated myofibroblasts further modify the ECM, increasing tissue stiffness and creating conditions that favor persistent activation rather than normal wound resolution [93,105].

Understanding this mechanical-biochemical integration offers potential therapeutic strategies targeting the mechanotransduction machinery alongside traditional anti-TGF-β approaches, potentially providing more effective interventions in fibrotic diseases [98,106].

## 4. Fibroblast–Immune Cell Interactions

**Figure 2:**
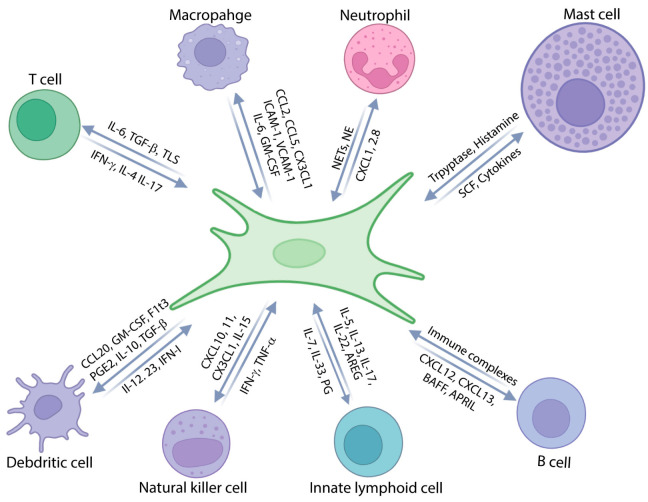
Fibroblasts engage in dynamic, bidirectional interactions with immune cells such as T cells, macrophages, neutrophils, mast cells, dendritic cells, natural killer cells, innate lymphoid cells, and B cells. These interactions both shape the immune response and are influenced by signals derived from immune cells.

The immunomodulatory functions of fibroblasts are realized largely through complex interactions with various immune cell populations, including neutrophils, mast cells, dendritic cells, natural killer cells, innate lymphoid cells, and B cells (Figure 2) [5,6,11,107,108]. These interactions are bidirectional, with fibroblasts both shaping immune responses and being influenced by immune cell-derived signals. These interactions collectively determine the nature and outcome of inflammatory responses in the tissues.

### 4.1. T Lymphocyte Interactions

Fibroblasts and T lymphocytes communicate both directly and through soluble factors, together shaping adaptive immune responses [109,110,111,112]. Co-culture experiments showing T cell-derived cytokines (IFN-γ, TNF-α) activate fibroblasts, which then produce factors modulating T cell survival [85]. Fibroblast-derived cytokines, like IL-6 and TGF-β, regulate T cell differentiation (notably, Th17 in psoriasis), while regulatory T cells (Tregs) can suppress fibroblast inflammatory function. T cell cytokines (IFN-γ, IL-4, IL-17, etc.) influence fibroblast behavior and ECM production [113,114]. Fibroblasts regulate T cell responses by depleting nutrients (e.g., tryptophan via IDO enzyme), and long-term contact between these cells supports T cell survival and memory. In chronic inflammation, fibroblasts help form tertiary lymphoid structures (TLS), supporting local immune responses.

### 4.2. Macrophage Interactions

Fibroblasts and macrophages interact bidirectionally to shape immunity and tissue remodeling [84,85]. Fibroblasts recruit macrophages using chemokines (CCL2, CCL5, CX3CL1), and their responses depend on macrophage type: pro-inflammatory M1 macrophages activate fibroblasts to produce inflammatory signals and degrade the ECM, while repair-promoting M2 macrophages stimulate fibroblast growth and collagen production for healing and fibrosis [115,116]. Direct co-culture experiments demonstrating fibroblasts and macrophages bidirectional signaling via IL-6, TNF-α, and TGF-β [117]. Prostate cancer-associated fibroblasts (CAFs) actively recruit monocytes to the tumor microenvironment, predominantly acting through CXCL12 delivery and subsequently promote their trans differentiation toward the M2 macrophage phenotype [118]. Cell–cell contact (via ICAM-1, VCAM-1, Notch) and cytokines (GM-CSF, IL-6) influence mutual activation and macrophage polarization. M1 macrophages may induce fibroblast death; M2 enhance survival. Fibroblasts and macrophages cooperation supports wound healing and angiogenesis but may also drive chronic inflammation or cancer [119]. Crosstalk between fibroblasts and macrophages shapes inflammation resolution and disrupted this coordination leads to chronic disease, such as impaired wound healing in older adults.

### 4.3. Neutrophil Interactions

Neutrophils are early responders in inflammation, recruited by fibroblast-produced chemokines like CXCL8, CXCL1, and CXCL2 [6,11,120]. Neutrophils activate fibroblasts via reactive oxygen species and proteases, such as neutrophil elastase (NE), triggering inflammatory signaling [121]. Neutrophil elastase knockout mice are protected from lung fibrosis by impairing TGF-β activation followed by inhibiting lung fibroblast proliferation and myofibroblast differentiation [122]. Neutrophil extracellular traps (NETs) also activate fibroblasts through receptors like TLRs, but can be cytotoxic as well. Fibroblasts influence NET formation and help clear NETs via DNase and phagocytosis [123]. Fibroblasts can also help resolve inflammation by engulfing apoptotic neutrophils, especially when macrophages are overwhelmed.

### 4.4. Mast Cell Interactions

Mast cells, key players in allergy and tissue repair, strongly interact with fibroblasts [124,125,126]. Direct co-culture experiments showing mast cell mediators induce human skin fibroblast proliferation and collagen synthesis, suggesting mast cells’ role in skin remodeling and fibrosis [127]. Upon activation, mast cells release mediators like histamine, tryptase, and cytokines that stimulate fibroblasts, affect their proliferation, cytokine production, and ECM synthesis. Mast cell tryptase stimulates both human dermal fibroblast proliferation and collagen production [127,128,129]. Fibroblasts, in turn, produce stem cell factor (SCF), support mast cell survival and localization, and release chemokines that attract mast cells. In fibrotic diseases, mast cell mediators (TGF-β, tryptase) further activate fibroblasts and drive pathological fibrosis, as seen in systemic sclerosis.

### 4.5. Dendritic Cell Interactions

Dendritic cells (DCs) link innate and adaptive immunity and interact extensively with fibroblasts [108,130]. Fibroblasts recruit and support DCs through chemokines (e.g., CCL20) and growth factors (GM-CSF, Flt3 ligand) [131]. DCs release cytokines (IL-12, IL-23, type I interferons) that alter fibroblast function and promote chemokine production for T cell recruitment [132]. Fibroblasts also regulate DC maturation and immune responses via mediators such as PGE2, IL-10, and TGF-β, promoting either effector or regulatory T cells [133]. In chronically inflamed skin, fibroblastic reticular cells cooperate with DCs to form tertiary lymphoid structures, facilitating DC-T cell interactions [111].

### 4.6. Natural Killer Cell Interactions

Natural killer (NK) cells are innate lymphocytes with cytotoxic and immunoregulatory functions [5,134]. The interaction between NK cells and fibroblasts has received less attention than other immune cell–fibroblast interactions but is increasingly recognized as important. Activated NK cells could kill mesenchymal stromal cells (MSCs), whereas MSCs strongly inhibited interleukin-2 (IL-2)-induced NK-cell proliferation [135]. This bidirectional interaction is context-dependent, with the balance determined by NK cell activation state, MSC-to-NK cell ratio, and microenvironmental factors. Cancer-associated fibroblasts in melanoma tumors protect malignant cells from NK cell-mediated killing by secreting MMPs that proteolytically cleave MICA and MICB from the tumor cell surface. This MMP-mediated shedding reduces membrane-bound NKG2D ligands, preventing effective NK cell activation through the NKG2D receptor and consequently impairing NK cell cytotoxicity against melanoma cells [136]. NK cells, known for cytotoxic and regulatory roles, interact with fibroblasts via chemokines (CXCL10, CXCL11, CX3CL1) and IL-15, which supports NK cell survival and activation. NK cells release IFN-γ and TNF-α to stimulate fibroblast inflammatory responses, and can kill activated fibroblasts, controlling fibroblast accumulation [137]. Fibroblasts may express ligands for NK cell receptors, allowing NK cells to target abnormal fibroblasts, with these interactions influenced by inflammatory conditions [138].

### 4.7. Innate Lymphoid Cell Interactions

Innate lymphoid cells (ILCs) and fibroblasts engage in bidirectional crosstalk that is crucial for tissue homeostasis, immune responses, and pathological conditions. This interaction occurs through both direct cell–cell contact and paracrine signaling mechanisms [139,140]. ILCs, which lack antigen-specific receptors, influence fibroblasts through cytokine production [141]. ILC2s release IL-5, IL-13, and amphiregulin, activating fibroblasts to increase collagen production and drive tissue repair or fibrosis [142,143,144]. ILC3s secrete IL-17 and IL-22, similarly activating fibroblasts, especially during early inflammation. Fibroblasts can also modulate ILC activity via IL-7, IL-33, and prostaglandins, showing bidirectional communication [143,145,146].

### 4.8. B Cell Interactions

B cell–fibroblast interactions contribute to inflammation, especially in autoimmune diseases [147,148]. Fibroblasts attract and organize B cells via CXCL12 and CXCL13 and support their survival anIL-17, antibody production in tertiary lymphoid structures [149]. B cells and fibroblasts interact in multiple immunological contexts, particularly in lymphoid organs. Fibroblastic reticular cells (FRCs)provide structural support and create niches for B cell survival and differentiation. CXCL13 production by FRCs attracts B cells to follicles, and BAFF (B cell activating factor) secretion by FRCs supports B cell survival [150]. B cell antibodies can activate fibroblasts through Fc receptors, driving inflammatory responses. Fibroblasts also produce BAFF and APRIL to promote B cell function [151].

Overall, fibroblasts influence and are influenced by a range of immune cells, such as neutrophils, macrophages, T and B lymphocytes, dendritic cells, mast cells, NK cells, and innate lymphoid cells, through both direct contact and secret factors. These bidirectional interactions shape inflammatory responses, tissue repair, fibrosis, and immunity.

## 5. Fibroblasts in Inflammatory Skin Diseases

**Figure 3:**
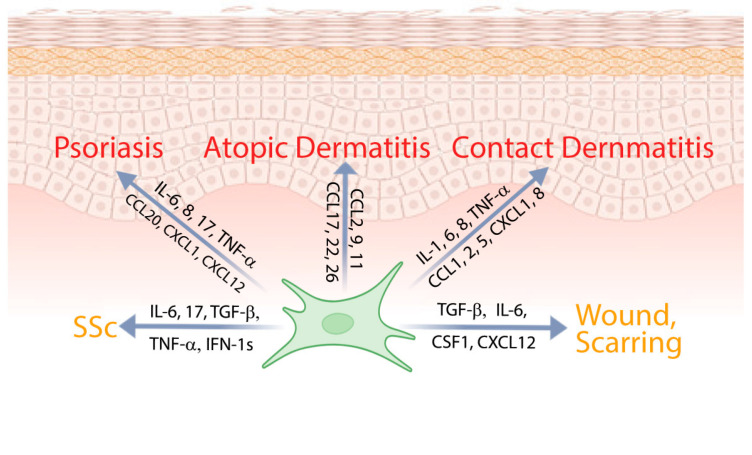
Fibroblasts play an important pathogenic role in various inflammatory skin diseases, including psoriasis, atopic dermatitis, contact dermatitis, systemic sclerosis, and in processes such as inflammatory wound healing and fibrotic scarring.

The involvement of fibroblasts in specific inflammatory skin diseases highlights their pathogenic significance and reveals disease-specific mechanisms (Figure 3) [6,14,152,153,154]. Understanding these context-dependent roles of fibroblasts not only illuminates their contribution to disease pathogenesis but also points to potential therapeutic targets aimed at modulating fibroblast function in inflammatory skin disorders.

### 5.1. Psoriasis

Psoriasis is a chronic inflammatory disease characterized by epidermal hyperplasia, immune cell infiltration, and angiogenesis [114,155]. Dermal fibroblasts contribute significantly to psoriasis pathogenesis through communication of keratinocytes [7,153,156]. Psoriatic fibroblasts exhibit enhanced responsiveness to IL-17 and TNF-α, producing elevated levels of IL-6, IL-8, CCL20, CXCL1, and CXCL12 [7,87,114,153,157]. Single-cell analyses have identified expanded populations of inflammatory fibroblasts in psoriatic lesions expressing genes associated with IL-17 and TNF-α signaling [153,158]. These fibroblasts recruit neutrophils and T cells through chemokine secretion and support keratinocyte proliferation. Additionally, psoriatic fibroblasts produce vascular endothelial growth factor (VEGF) that promotes pathological angiogenesis characteristic of psoriasis [159]. Therapeutic targeting of TNF-α and IL-17 in psoriasis likely affects not only immune cells but also dermal fibroblasts, disrupting the inflammatory networks that sustain disease. Understanding fibroblast contributions to psoriasis may enable development of more selective therapeutic interventions.

### 5.2. Atopic Dermatitis

Atopic dermatitis (AD) is a chronic inflammatory skin disease driven predominantly by Th2 immune responses and characterized by barrier dysfunction, pruritus, and eczematous lesions [160,161]. Dermal fibroblasts in AD lesions respond to Th2 cytokines IL-4 and IL-13, producing chemokines such as CCL17, CCL22, and CCL26 that recruit Th2 cells and eosinophils, thereby amplifying type 2 inflammation [153]. AD fibroblasts also produce thymic stromal lymphopoietin (TSLP), which activates dendritic cells and promotes Th2 differentiation. Additionally, these fibroblasts exhibit altered ECM production, with decreased expression of filaggrin-degradation products and antimicrobial peptides that compromise barrier function. The interaction between fibroblasts and keratinocytes is particularly important in AD, as fibroblast-derived factors influence keratinocyte differentiation, lipid metabolism, and inflammatory responses [162].

### 5.3. Systemic Sclerosis and Scleroderma

Systemic sclerosis (SSc) is an autoimmune connective tissue disease characterized by fibrosis of skin and internal organs, vascular dysfunction, and immune dysregulation [163]. Fibroblasts are central to SSc pathogenesis, exhibiting persistent activation, excessive collagen synthesis, and myofibroblast differentiation driven by TGF-β, IL-6, and other profibrotic mediators [70,164]. SSc fibroblasts demonstrate intrinsic abnormalities including constitutive activation of profibrotic signaling pathways, resistance to apoptosis, and epigenetic alterations that sustain their pathological phenotype [165]. These cells produce excessive amounts of type I and type III collagen, fibronectin, and other ECM components while secreting inflammatory cytokines (TNF-α, IFN-1s, IL-17) that recruit immune cells. Subsets of SSc fibroblasts have been identified that express markers of inflammatory activation alongside profibrotic programs, suggesting that inflammation and fibrosis are interconnected processes [70,166].

### 5.4. Contact Dermatitis

Contact dermatitis, encompassing both allergic and irritant forms, involves acute inflammatory responses to external antigens or irritants [167,168]. Dermal fibroblasts participate in contact dermatitis by sensing irritants and allergens through TLRs and other receptors, subsequently producing inflammatory mediators (IL-1, IL-6, IL-8, TNF-α, CCL2, CCL5, CXCL1, CXCL8) that recruit and activate T cells and innate immune cells [168,169]. In allergic contact dermatitis, hapten-specific T cells interact with antigen-presenting cells and potentially with fibroblasts expressing MHC class II molecules [170]. Fibroblast-derived chemokines establish inflammatory milieu conducive to T cell infiltration, while fibroblast production of IL-6 and prostaglandins contributes to the clinical manifestations of inflammation including erythema, edema, and pruritus.

### 5.5. Chronic Wounds and Fibrotic Scarring

Chronic non-healing wounds and pathological scarring represent conditions where dysregulated fibroblast function perpetuates inflammation [171,172]. In chronic wounds, fibroblasts exhibit senescent phenotypes, impaired proliferation, and altered responsiveness to growth factors, contributing to failure of wound closure [173]. The persistent inflammatory environment in chronic wounds, characterized by elevated levels of MMPs, inflammatory cytokines, and bacterial colonization, sustains dysfunctional fibroblast behavior. Conversely, in fibrotic scarring, excessive fibroblast activation and prolonged inflammatory signaling lead to pathological ECM accumulation [174]. Keloid fibroblasts demonstrate increased proliferation, resistance to apoptosis, and elevated production of collagen and inflammatory mediators including IL-6 and TGF-β [175]. Adult mammalian skin wounds generally heal with fibrotic scars, which involve the deposition of excess abnormally organized, densely packed collagen fibrils, a hallmark of the fibrotic scar. Recent report demonstrates that injured reindeer antler velvet skin regenerates without a scar, while injured reindeer back skin forms a fibrotic scar [88]. Single-cell RNA sequencing and proteomics identified that fibroblasts from antler skin wounds displayed distinct inflammatory phenotypes/signatures resembling developmental and regenerative characteristics like human fetal fibroblasts. In contrast, fibroblasts from the back skin of reindeer demonstrate a transcriptional program associated with pro-inflammatory responses. Specifically, fibroblast-derived cytokines, CSF1 and CXCL12, function as master mediators of inflammatory priming and direct site-specific immune cell recruitment to promote scar formation. These findings indicate that fibroblasts regulate immune responses to wounding, influencing wound regeneration and fibrotic scarring.

Fibroblasts play a key pathogenic role in a range of inflammatory skin diseases, exhibiting disease-specific functions and mechanisms that both drive and sustain inflammation and tissue remodeling. Overall, understanding the diverse and context-specific activities of fibroblasts not only clarifies their pivotal contributions to skin disease pathogenesis, but also reveals promising therapeutic targets to modulate fibroblast function and improve disease outcomes.

## 6. Therapeutic Implications

**Figure 4:**
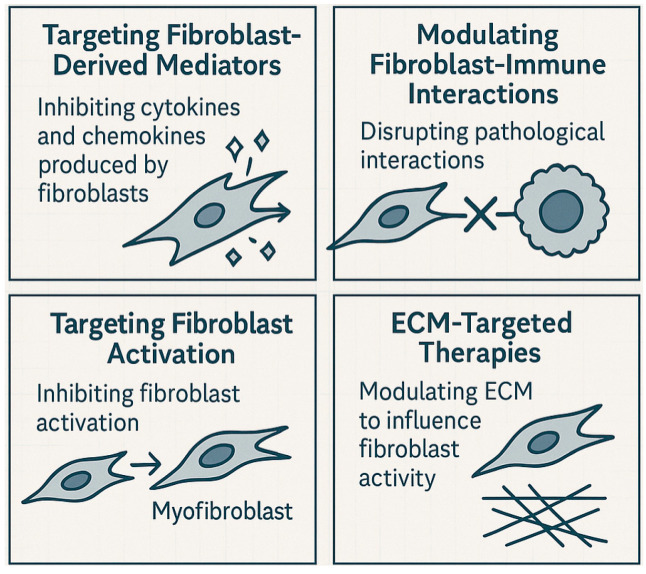
Therapeutic strategies targeting fibroblasts, such as inhibiting fibroblast-derived mediators, modulating fibroblast–immune interactions, suppressing pathological activation, and manipulating extracellular matrix properties, represent promising approaches for the treatment of inflammatory diseases.

The evolving understanding of fibroblasts as dynamic inflammatory mediators rather than simply structural support cells marks a fundamental transformation in how we conceptualize inflammatory disease mechanisms and has opened up new therapeutic horizons (Figure 4). Most dermal fibroblasts possess intrinsic immunomodulatory machinery (pattern recognition receptors, cytokine receptors, immune signaling components), but activation depends on the tissue microenvironment and disease context. As such, immunomodulatory fibroblast populations represent context-dependent functional states rather than universal or disease-specific subsets. Targeting fibroblasts offers distinct advantages over conventional immune cell-directed therapies: a single intervention can simultaneously interrupt multiple disease-perpetuating pathways and address the persistent tissue alterations that underlie chronicity. Moreover, because fibroblasts are long-lived tissue residents, therapeutic modulation of these cells may produce sustained clinical responses, potentially overcoming the relapsing pattern characteristic of many inflammatory conditions.

There is substantial literature on the therapeutic use of fibroblast-based cell therapies. These approaches have been established across diverse clinical applications, including cosmetic procedures (wrinkle reduction) [176,177], wound healing (diabetic and pressure ulcers) [178,179], and tissue engineering (cartilage and ligament repair) [180,181]. While these applications demonstrate the versatility of fibroblasts in regenerative medicine, their direct use in immunotherapy remains relatively limited compared to other cell types such as T cells, dendritic cells, and mesenchymal stem cells.

### 6.1. Targeting Fibroblast-Derived Mediators

A promising therapeutic strategy involves inhibiting key cytokines and chemokines produced by fibroblasts, which contribute to the pathogenesis of various inflammatory skin diseases. Biologics targeting IL-6, IL-17, and TNF-α have demonstrated efficacy in psoriasis, and their effects likely extend to fibroblast populations [182]. Similarly, targeting TSLP and IL-4/IL-13 signaling in atopic dermatitis may suppress fibroblast contributions to type 2 inflammation [183]. Small molecule inhibitors of JAK-STAT, NF-κB, and MAPK signaling pathways can modulate fibroblast inflammatory responses and are under investigation for multiple inflammatory dermatoses [184]. Additionally, targeting specific chemokine receptors expressed by immune cells could disrupt fibroblast-mediated immune cell recruitment. These approaches highlight the potential of targeting fibroblast-derived mediators, both extracellularly and intracellularly, to disrupt the immunopathogenic cycle driving chronic skin inflammation.

### 6.2. Modulating Fibroblast–Immune Interactions

Strategies that disrupt pathological fibroblast–immune cell interactions while preserving homeostatic functions represent an attractive therapeutic approach [8,185]. Blocking specific adhesion molecules or co-stimulatory pathways on fibroblasts could reduce excessive T cell activation without causing broad immunosuppression [186]. Cellular therapies involving modification of fibroblast phenotypes or transplantation of regulatory fibroblast populations are emerging concepts.

### 6.3. Targeting Fibroblast Activation

In fibrotic inflammatory conditions such as systemic sclerosis, inhibiting fibroblast activation and myofibroblast differentiation represents a primary therapeutic goal. Antifibrotic agents targeting TGF-β signaling, including pirfenidone and nintedanib, have shown efficacy in pulmonary fibrosis and are being evaluated for cutaneous fibrosis [187]. Senolytic agents that selectively eliminate senescent fibroblasts or senostatic drugs that suppress the SASP may reduce chronic inflammation and improve tissue homeostasis in aging and chronic inflammatory conditions [188].

### 6.4. ECM-Targeted Therapies

Modulation of ECM composition and mechanical properties represents an indirect approach to influencing fibroblast mechanical properties and behavior [172]. MMP inhibitors can reduce excessive matrix degradation in certain contexts, while controlled delivery of ECM-degrading enzymes can promote remodeling of pathological scars [189]. Additionally, inhibitors of lysyl oxidase-like 2 (LOXL2), which crosslinks collagen, may reduce tissue stiffness and interrupt mechanotransduction-mediated fibroblast activation [190]. Biomaterials and scaffolds designed to recapitulate normal ECM properties may reprogram fibroblast phenotypes toward homeostatic states. Furthermore, targeting specific matricellular proteins or ECM fragments that serve as DAMPs could dampen inflammation propagated through ECM–fibroblast interactions. Hyaluronan oligosaccharides, decorin, and other bioactive ECM fragments have shown potential in preclinical studies [28,29,30,31,32].

However, therapeutic targeting of fibroblasts requires careful consideration of their essential homeostatic functions in maintaining tissue structure and integrity. Disrupting these homeostatic functions through fibroblast-targeted therapies can produce significant unintended consequences, including impaired wound healing and tissue regeneration, ECM structural instability, dysregulated immune responses, and loss of stem cell niche support.

Effective therapeutic strategies must therefore be selective, suppressing pathological fibroblast activities while preserving their beneficial roles in tissue maintenance and ECM homeostasis. This selectivity can be achieved through several strategic approaches: targeting pathologically activated fibroblast subpopulations, inhibiting disease-promoting signaling pathways while preserving physiological ones, restricting interventions to periods of active disease, or leveraging fibroblast heterogeneity to reprogram rather than eliminate these cells. Central to achieving this selectivity is understanding fibroblast heterogeneity, which is crucial for deciphering their roles in skin inflammation. Different fibroblast subsets exhibit varying capacities to respond to inflammatory stimuli, produce specific mediators, and interact with immune cells. Identifying which fibroblast populations drive pathological inflammation in different disease contexts will enable more precise therapeutic interventions that selectively target disease-promoting activities while sparing homeostatic functions.

## 7. Conclusions and Future Directions

Fibroblasts have emerged as central orchestrators of tissue inflammation, functioning far beyond their traditional structural roles. These versatile cells sense inflammatory stimuli through diverse PRRs and cytokine receptors, produce an extensive repertoire of inflammatory mediators, engage in complex bidirectional interactions with immune cells, and dynamically remodel the ECM in response to inflammatory signals.

The involvement of dermal fibroblasts in inflammatory skin diseases including psoriasis, atopic dermatitis, systemic sclerosis, contact dermatitis, and wound healing disorders underscores their pathogenic significance. Dysregulated fibroblast functions contribute to disease initiation, perpetuation, and chronicity through sustained production of inflammatory mediators, recruitment and activation of immune cells, and pathological tissue remodeling. Understanding the immunomodulatory roles of dermal fibroblasts provides important insights into skin pathology and reveals numerous therapeutic opportunities. Targeting fibroblast-derived mediators, modulating fibroblast–immune interactions, inhibiting pathological fibroblast activation, and manipulating ECM properties represent promising strategies for treating inflammatory dermatoses. As technologies advance and our understanding deepens, fibroblast-directed therapies may achieve more precise control of cutaneous inflammation with improved efficacy and safety profiles.

Future research should focus on comprehensive characterization of fibroblast heterogeneity in health and disease, elucidation of molecular mechanisms governing fibroblast activation and resolution, identification of fibroblast-specific therapeutic targets, and translation of mechanistic insights into clinical applications. Translating knowledge of immunomodulatory fibroblasts into clinical practice may include developing drugs or biologics that selectively inhibit pro-inflammatory fibroblast activity or enhance their reparative functions. The integration of advanced technologies including single-cell omics, spatial transcriptomics, lineage tracing, and ex vivo modeling will accelerate progress toward these goals. Ultimately, recognizing fibroblasts as dynamic immunomodulators rather than passive structural cells fundamentally reshapes our understanding of tissue inflammation and offers hope for improved therapeutic strategies.

## Data Availability

No new data were created or analyzed in this study.

## References

[1] Tracy L.E., Minasian R.A., Caterson E.J. (2016). Extracellular matrix and dermal fibroblast function in the healing wound. Adv. Wound Care.

[2] Plikus M.V., Wang X., Sinha S., Forte E., Thompson S.M., Herzog E.L., Driskell R.R., Rosenthal N., Biernaskie J., Horsley V. (2021). Fibroblasts: Origins, definitions, and functions in health and disease. Cell.

[3] Davidson S., Coles M., Thomas T., Kollias G., Ludewig B., Turley S., Brenner M., Buckley C.D. (2021). Fibroblasts as immune regulators in infection, inflammation and cancer. Nat. Rev. Immunol..

[4] Zou A.E., Kongthong S., Mueller A.A., Brenner M.B. (2025). Fibroblasts in immune responses, inflammatory diseases and therapeutic implications. Nat. Rev. Rheumatol..

[5] Xiao Z., Pure E. (2025). The fibroinflammatory response in cancer. Nat. Rev. Cancer.

[6] Cavagnero K.J., Gallo R.L. (2022). Essential immune functions of fibroblasts in innate host defense. Front. Immunol..

[7] Ma F.Y., Plazyo O., Billi A.C., Tsoi L.C., Xing X.Y., Wasikowski R., Gharaee-Kermani M., Hile G., Jiang Y.Y., Harms P.W. (2023). Single cell and spatial sequencing define processes by which keratinocytes and fibroblasts amplify inflammatory responses in psoriasis. Nat. Commun..

[8] Chu C.Q., Quan T.H. (2024). Fibroblast yap/taz signaling in extracellular matrix homeostasis and tissue fibrosis. J. Clin. Med..

[9] Kalluri R. (2016). The biology and function of fibroblasts in cancer. Nat. Rev. Cancer.

[10] Lynch M.D., Watt F.M. (2018). Fibroblast heterogeneity: Implications for human disease. J. Clin. Investig..

[11] Cavagnero K.J., Li F., Dokoshi T., Nakatsuji T., O’Neill A.M., Aguilera C., Liu E., Shia M., Osuoji O., Hata T. (2024). CXCL12+ dermal fibroblasts promote neutrophil recruitment and host defense by recognition of IL-17. J. Exp. Med..

[12] Ko K.I., Merlet J.J., DerGarabedian B.P., Zhen H., Suzuki-Horiuchi Y., Hedberg M.L., Hu E., Nguyen A.T., Prouty S., Alawi F. (2022). Nf-κB perturbation reveals unique immunomodulatory functions in prx1^+^ fibroblasts that promote development of atopic dermatitis. Sci. Transl. Med..

[13] Bensa T., Tekkela S., Rognoni E. (2023). Skin fibroblast functional heterogeneity in health and disease. J. Pathol..

[14] Wei K.V., Nguyen H.N., Brenner M.B. (2021). Fibroblast pathology in inflammatory diseases. J. Clin. Investig..

[15] Piccinini A.M., Midwood K.S. (2010). Dampening inflammation by modulating tlr signalling. Mediat. Inflamm..

[16] Schaefer L. (2014). Complexity of danger: The diverse nature of damage-associated molecular patterns. J. Biol. Chem..

[17] Kawai T., Akira S. (2010). The role of pattern-recognition receptors in innate immunity: Update on toll-like receptors. Nat. Immunol..

[18] Pierer M., Rethage J., Seibl R., Lauener R., Brentano F., Wagner U., Hantzschel H., Michel B.A., Gay R.E., Gay S. (2004). Chemokine secretion of rheumatoid arthritis synovial fibroblasts stimulated by toll-like receptor 2 ligands. J. Immunol..

[19] Bombardieri M., Kam N.W., Brentano F., Choi K., Filer A., Kyburz D., McInnes I.B., Gay S., Buckley C., Pitzalis C. (2011). A BAFF/APRIL-dependent TLR_3_-stimulated pathway enhances the capacity of rheumatoid synovial fibroblasts to induce AID expression and Ig class-switching in B cells. Ann. Rheum. Dis..

[20] Brentano F., Schorr O., Gay R.E., Gay S., Kyburz D. (2005). Rna released from necrotic synovial fluid cells activates rheumatoid arthritis synovial fibroblasts via toll-like receptor 3. Arthritis Rheum..

[21] Bautista-Hernandez L.A., Gomez-Olivares J.L., Buentello-Volante B., Bautista-de Lucio V.M. (2017). Fibroblasts: The unknown sentinels eliciting immune responses against microorganisms. Eur. J. Microbiol. Immunol..

[22] Wang J., Hori K., Ding J., Huang Y., Kwan P., Ladak A., Tredget E.E. (2011). Toll-like receptors expressed by dermal fibroblasts contribute to hypertrophic scarring. J. Cell Physiol..

[23] Yao C., Oh J.H., Lee D.H., Bae J.S., Jin C.L., Park C.H., Chung J.H. (2015). Toll-like receptor family members in skin fibroblasts are functional and have a higher expression compared to skin keratinocytes. Int. J. Mol. Med..

[24] Sameer A.S., Nissar S. (2021). Toll-like receptors (tlrs): Structure, functions, signaling, and role of their polymorphisms in colorectal cancer susceptibility. BioMed Res. Int..

[25] Koliaraki V., Chalkidi N., Henriques A., Tzaferis C., Polykratis A., Waisman A., Muller W., Hackam D.J., Pasparakis M., Kollias G. (2019). Innate sensing through mesenchymal TLR4/MyD88 signals promotes spontaneous intestinal tumorigenesis. Cell Rep..

[26] Schnittker D., Kwofie K., Ashkar A., Trigatti B., Richards C.D. (2013). Oncostatin m and TLR-4 ligand synergize to induce MCP-1, IL-6, and VEGF in human aortic adventitial fibroblasts and smooth muscle cells. Mediat. Inflamm..

[27] Franchi L., Eigenbrod T., Muñoz-Planillo R., Nuñez G. (2009). The inflammasome: A caspase-1-activation platform that regulates immune responses and disease pathogenesis. Nat. Immunol..

[28] Garantziotis S., Brezina M., Castelnuovo P., Drago L. (2016). The role of hyaluronan in the pathobiology and treatment of respiratory disease. Am. J. Physiol. Lung Cell Mol. Physiol..

[29] Teder P., Vandivier R.W., Jiang D., Liang J., Cohn L., Pure E., Henson P.M., Noble P.W. (2002). Resolution of lung inflammation by CD44. Science.

[30] Schaefer L., Babelova A., Kiss E., Hausser H., Marsche G., Young M., Götte M., Malle E., Schaefer R.M., Gröne H.J. (2005). The matrix component biglycan is proinflammatory and signals through toll-like receptor-4 and -2 in macrophages. J. Clin. Investig..

[31] Merline R., Moreth K., Beckmann J., Nastase M.V., Zeng-Brouwers J., Tralhao J.G., Lemarchand P., Pfeilschifter J., Schaefer R.M., Iozzo R.V. (2011). Signaling by the matrix proteoglycan decorin controls inflammation and cancer through PDCD4 and MicroRNA-21. Sci. Signal..

[32] Okamura Y., Watari M., Jerud E.S., Young D.W., Ishizaka S.T., Rose J., Chow J.C., Strauss J. (2001). The extra domain A of fibronectin activates toll-like receptor 4. J. Biol. Chem..

[33] Scaffidi P., Misteli T., Bianchi M.E. (2002). Release of chromatin protein hmgb1 by necrotic cells triggers inflammation. Nature.

[34] Turner N.A. (2016). Inflammatory and fibrotic responses of cardiac fibroblasts to myocardial damage associated molecular patterns (DAMPs). J. Mol. Cell. Cardiol..

[35] Ospelt C., Brentano F., Rengel Y., Stanczyk J., Kolling C., Tak P.P., Gay R.E., Gay S., Kyburz D. (2008). Overexpression of toll-like receptors 3 and 4 in synovial tissue from patients with early rheumatoid arthritis toll-like receptor expression in early and longstanding arthritis. Arthritis Rheum..

[36] Yoshitomi H. (2019). Regulation of immune responses and chronic inflammation by fibroblast-like synoviocytes. Front. Immunol..

[37] Debets R., Hegmans J.P., Deleuran M., Hooft S., Benner R., Prens E.P. (1996). Expression of cytokines and their receptors by psoriatic fibroblast. I. Altered IL-6 synthesis. Cytokine.

[38] Sims J.E., Smith D.E. (2010). The IL-1 family: Regulators of immunity. Nat. Rev. Immunol..

[39] Weber A., Wasiliew P., Kracht M. (2010). Interleukin-1beta (IL-1beta) processing pathway. Sci. Signal..

[40] Onishi R.M., Gaffen S.L. (2010). Interleukin-17 and its target genes: Mechanisms of interleukin-17 function in disease. Immunology.

[41] Hwang S.Y., Kim J.Y., Kim K.W., Park M.K., Moon Y., Kim W.U., Kim H.Y. (2004). IL-17 induces production of IL-6 and IL-8 in rheumatoid arthritis synovial fibroblasts via NF-κB- and PI3-kinase/Akt-dependent pathways. Arthritis Res. Ther..

[42] Berman B., Wietzerbin J. (1992). Tumor necrosis factor-α (TNF-α), interferon-α (INF-α) and interferon-γ (INF-γ) receptors on human normal and scleroderma dermal fibroblasts in vitro. J. Dermatol. Sci..

[43] Horiuchi M., Hayashida W., Akishita M., Yamada S., Lehtonen J.Y., Tamura K., Daviet L., Chen Y.E., Hamai M., Cui T.X. (2000). Interferon-γ induces AT_2_ receptor expression in fibroblasts by Jak/STAT pathway and interferon regulatory factor-1. Circ. Res..

[44] Platanias L.C. (2005). Mechanisms of type-I- and type-II-interferon-mediated signalling. Nat. Rev. Immunol..

[45] Darnell J.E., Kerr I.M., Stark G.R. (1994). Jak-stat pathways and transcriptional activation in response to IFNS and other extracellular signaling proteins. Science.

[46] Eloranta M.L., Franck-Larsson K., Lovgren T., Kalamajski S., Ronnblom A., Rubin K., Alm G.V., Ronnblom L. (2010). Type I interferon system activation and association with disease manifestations in systemic sclerosis. Ann. Rheum. Dis..

[47] Crow M.K. (2014). Type I interferon in the pathogenesis of lupus. J. Immunol..

[48] Saraiva M., O’Garra A. (2010). The regulation of IL-10 production by immune cells. Nat. Rev. Immunol..

[49] Li D.Q., Tseng S.C.G. (1996). Differential regulation of cytokine and receptor transcript expression in human corneal and limbal fibroblasts by epidermal growth factor, transforming growth factor-alpha, platelet-derived growth factor B, and interleukin-1 beta. Investig. Ophthtalmol. Vis. Sci..

[50] Louis I.V., Bohjanen P.R. (2017). Post-transcriptional regulation of cytokine and growth factor signaling in cancer. Cytokine Growth Factor Rev..

[51] Tanaka T., Narazaki M., Kishimoto T. (2014). IL-6 in inflammation, immunity, and disease. Cold Spring Harb. Perspect. Biol..

[52] Brenner D., Blaser H., Mak T.W. (2015). Regulation of tumour necrosis factor signalling: Live or let die. Nat. Rev. Immunol..

[53] Quan T., Qin Z., Robichaud P., Voorhees J.J., Fisher G.J. (2011). CCN1 contributes to skin connective tissue aging by inducing age-associated secretory phenotype in human skin dermal fibroblasts. J. Cell Commun. Signal..

[54] Nguyen H.N., Noss E.H., Mizoguchi F., Huppertz C., Wei K.S., Watts G.F.M., Brenner M.B. (2017). Autocrine loop involving IL-6 family member LIF, LIF receptor, and STAT4 drives sustained fibroblast production of inflammatory mediators. Immunity.

[55] Paquet P., Pierard G.E. (1996). Interleukin-6 and the skin. Int. Arch. Allergy Immunol..

[56] Russo R.C., Garcia C.C., Teixeira M.M., Amaral F.A. (2014). The CXCL8/IL-8 chemokine family and its receptors in inflammatory diseases. Expert Rev. Clin. Immunol..

[57] Dinarello C.A. (2018). Overview of the IL-1 family in innate inflammation and acquired immunity. Immunol. Rev..

[58] Smith R.S., Smith T.J., Blieden T.M., Phipps R.P. (1997). Fibroblasts as sentinel cells—Synthesis of chemokines and regulation of inflammation. Am. J. Pathol..

[59] Mauviel A., Temime N., Charron D., Loyau G., Pujol J.P. (1991). Induction of interleukin-1-β production in human dermal fibroblasts by interleukin-1-α and tumor-necrosis-factor-α—Involvement of protein kinase-dependent and adenylate cyclase-dependent regulatory pathways. J. Cell Biochem..

[60] Lappalainen U., Whitsett J.A., Wert S.E., Tichelaar J.W., Bry K. (2005). Interleukin-1β causes pulmonary inflammation, emphysema, and airway remodeling in the adult murine lung. Am. J. Resp. Cell Mol..

[61] Chen C.J., Kono H., Golenbock D., Reed G., Akira S., Rock K.L. (2007). Identification of a key pathway required for the sterile inflammatory response triggered by dying cells. Nat. Med..

[62] Arend W.P., Malyak M., Guthridge C.J., Gabay C. (1998). Interleukin-1 receptor antagonist: Role in biology. Annu. Rev. Immunol..

[63] Ouyang W., Rutz S., Crellin N.K., Valdez P.A., Hymowitz S.G. (2011). Regulation and functions of the IL-10 family of cytokines in inflammation and disease. Annu. Rev. Immunol..

[64] Hamilton J.A., Piccoli D.S., Cebon J., Layton J.E., Rathanaswani P., Mccoll S.R., Leizer T. (1992). Cytokine regulation of colony-stimulating factor (CSF) production in cultured human synovial fibroblasts. 2. Similarities and differences in the control of interleukin-1 induction of granulocyte-macrophage CSF and granulocyte-CSF production. Blood.

[65] Leizer T., Cebon J., Layton J.E., Hamilton J.A. (1990). Cytokine regulation of colony-stimulating factor production in cultured human synovial fibroblasts. 1. Induction of GM-CSF and G-CSF production by interleukin-1 and tumor-necrosis-factor. Blood.

[66] Hamilton J.A. (2008). Colony-stimulating factors in inflammation and autoimmunity. Nat. Rev. Immunol..

[67] Ambler D.R., Rizk N.N., Jiang Z.L., Saed G.M., Diamond M.P. (2006). TNF-alpha expression in human normal peritoneal and adhesion fibroblasts: Regulation by hypoxia. Fertil. Steril..

[68] Jordana M., Sarnstrand B., Sime P.J., Ramis I. (1994). Immune-inflammatory functions of fibroblasts. Eur. Respir. J..

[69] Armaka M., Konstantopoulos D., Tzaferis C., Lavigne M.D., Sakkou M., Liakos A., Sfikakis P.P., Dimopoulos M.A., Fousteri M., Kollias G. (2022). Single-cell multimodal analysis identifies common regulatory programs in synovial fibroblasts of rheumatoid arthritis patients and modeled TNF-driven arthritis. Genome Med..

[70] Worrell J.C., O’Reilly S. (2020). Bi-directional communication: Conversations between fibroblasts and immune cells in systemic sclerosis. J. Autoimmun..

[71] Liu X.T., Fang S.C., Liu H.J., Wang X.G., Dai X.N., Yin Q., Yun T.W., Wang W., Zhang Y.M., Liao H. (2015). Role of human pulmonary fibroblast-derived MCP-1 in cell activation and migration in experimental silicosis. Toxicol. Appl. Pharm..

[72] Yamamoto T., Eckes B., Hartmann K., Krieg T. (2001). Expression of monocyte chemoattractant protein-1 in the lesional skin of systemic sclerosis. J. Dermatol. Sci..

[73] Deshmane S.L., Kremlev S., Amini S., Sawaya B.E. (2009). Monocyte chemoattractant protein-1 (MCP-1): An overview. J. Interferon Cytokine Res..

[74] Malhotra D., Fletcher A.L., Astarita J., Lukacs-Kornek V., Tayalia P., Gonzalez S.F., Elpek K.G., Chang S.K., Knoblich K., Hemler M.E. (2012). Transcriptional profiling of stroma from inflamed and resting lymph nodes defines immunological hallmarks. Nat. Immunol..

[75] Schutyser E., Struyf S., Van Damme J. (2003). The CC chemokine CCL20 and its receptor CCR6. Cytokine Growth Factor Rev..

[76] Strieter R.M., Phan S.H., Showell H.J., Remick D.G., Lynch J.P., Genord M., Raiford C., Eskandari M., Marks R.M., Kunkel S.L. (1989). Monokine-induced neutrophil chemotactic factor gene-expression in human-fibroblasts. J. Biol. Chem..

[77] Hou S.M., Chen P.C., Lin C.M., Fang M.L., Chi M.C., Liu J.F. (2020). CXCL1 contributes to IL-6 expression in osteoarthritis and rheumatoid arthritis synovial fibroblasts by CXCR2, c-Raf, MAPK, and AP-1 pathway. Arthritis Res. Ther..

[78] Lukacs N.W., Chensue S.W., Smith R.E., Strieter R.M., Warmington K., Wilke C., Kunkel S.L. (1994). Production of monocyte chemoattractant protein-1 and macrophage inflammatory protein-1 alpha by inflammatory granuloma fibroblasts. Am. J. Pathol..

[79] Dragulev B., Bao Y.D., Ramos-Cerrillo B., Vazquez H., Olvera A., Stock R., Algaron A., Fox J.W. (2007). Upregulation of IL-6, IL-8, CXCL_1_, and CXCL_2_ dominates gene expression in human fibroblast cells exposed to loxosceles reclusa sphingomyelinase D: Insights into spider venom dermonecrosis. J. Investig. Dermatol..

[80] Worrell J.C., Walsh S.M., Fabre A., Kane R., Hinz B., Keane M.P. (2020). CXCR3A promotes the secretion of the antifibrotic decoy receptor sIL-13Rα2 by pulmonary fibroblasts. Am. J. Physiol.-Cell Physiol..

[81] Zlotnik A., Yoshie O. (2012). The chemokine superfamily revisited. Immunity.

[82] Rajarathnam K., Schnoor M., Richardson R.M., Rajagopal S. (2019). How do chemokines navigate neutrophils to the target site: Dissecting the structural mechanisms and signaling pathways. Cell. Signal..

[83] Ho T.K., Shiwen X., Abraham D., Tsui J., Baker D. (2012). Stromal-cell-derived factor-1 (SDF-1)/CXCL12 as potential target of therapeutic angiogenesis in critical leg ischaemia. Cardiol. Res. Pract..

[84] Timperi E., Gueguen P., Molgora M., Magagna I., Kieffer Y., Lopez-Lastra S., Sirven P., Baudrin L.G., Baulande S., Nicolas A. (2022). Lipid-associated macrophages are induced by cancer-associated fibroblasts and mediate immune suppression in breast cancer. Cancer Res..

[85] Buckley C.D., Pilling D., Lord J.M., Akbar A.N., Scheel-Toellner D., Salmon M. (2001). Fibroblasts regulate the switch from acute resolving to chronic persistent inflammation. Trends Immunol..

[86] Nagasawa T. (2014). Cxc chemokine ligand 12 (CXCL12) and its receptor CXCR4. J. Mol. Med..

[87] Quan C., Cho M.K., Shao Y., Mianecki L.E., Liao E., Perry D., Quan T. (2015). Dermal fibroblast expression of stromal cell-derived factor-1 (SDF-1) promotes epidermal keratinocyte proliferation in normal and diseased skin. Protein Cell.

[88] Sinha S., Sparks H.D., Labit E., Robbins H.N., Gowing K., Jaffer A., Kutluberk E., Arora R., Raredon M.S.B., Cao L. (2022). Fibroblast inflammatory priming determines regenerative versus fibrotic skin repair in reindeer. Cell.

[89] Quan T., Fisher G.J. (2015). Role of age-associated alterations of the dermal extracellular matrix microenvironment in human skin aging: A mini-review. Gerontology.

[90] Quan T., Wang F., Shao Y., Rittie L., Xia W., Orringer J.S., Voorhees J.J., Fisher G.J. (2013). Enhancing structural support of the dermal microenvironment activates fibroblasts, endothelial cells, and keratinocytes in aged human skin in vivo. J. Investig. Dermatol..

[91] Willenborg S., Schönborn K., Sawant M., Bornikoel A., Yamane T., Zeinert I., Eckes B., Eming S.A., Krieg T. (2025). Fibroblast-derived TGFβ1 regulates skin repair and fibrosis. Wound Repair Regen..

[92] Hinz B. (2009). Tissue stiffness, latent TGF-β1 activation, and mechanical signal transduction: Implications for the pathogenesis and treatment of fibrosis. Curr. Rheumatol. Rep..

[93] Hinz B. (2015). The extracellular matrix and transforming growth factor-β1: Tale of a strained relationship. Matrix Biol..

[94] Flavell R.A., Sanjabi S., Wrzesinski S.H., Licona-Limon P. (2010). The polarization of immune cells in the tumour environment by TGFβ. Nat. Rev. Immunol..

[95] Chan H., Li F.W., Dokoshi T., Cavagnero K.J., Li Q., Chen Y., Aguilera C., Nakatsuji T., Liu E.D., Indra A. (2025). Psychological stress increases skin infection through the action of TGFβ to suppress immune-acting fibroblasts. Sci. Immunol..

[96] Hinz B. (2007). Formation and function of the myofibroblast during tissue repair. J. Investig. Dermatol..

[97] Tomasek J.J., Gabbiani G., Hinz B., Chaponnier C., Brown R.A. (2002). Myofibroblasts and mechano-regulation of connective tissue remodelling. Nat. Rev. Mol. Cell Biol..

[98] Hinz B. (2010). The myofibroblast: Paradigm for a mechanically active cell. J. Biomech..

[99] Hinz B., Celetta G., Tomasek J.J., Gabbiani G., Chaponnier C. (2001). Alpha-smooth muscle actin expression upregulates fibroblast contractile activity. Mol. Biol. Cell.

[100] Wipff P.J., Rifkin D.B., Meister J.J., Hinz B. (2007). Myofibroblast contraction activates latent TGF-β1 from the extracellular matrix. J. Cell Biol..

[101] Balestrini J.L., Chaudhry S., Sarrazy V., Koehler A., Hinz B. (2012). The mechanical memory of lung myofibroblasts. Integr. Biol..

[102] Hinz B. (2013). It has to be the αv: Myofibroblast integrins activate latent TGF-β1. Nat. Med..

[103] Gabbiani G. (2003). The myofibroblast in wound healing and fibrocontractive diseases. J. Pathol..

[104] Hinz B., Phan S.H., Thannickal V.J., Galli A., Bochaton-Piallat M.L., Gabbiani G. (2007). The myofibroblast—One function, multiple origins. Am. J. Pathol..

[105] Thannickal V.J., Zhou Y., Gaggar A., Duncan S.R. (2014). Fibrosis: Ultimate and proximate causes. J. Clin. Investig..

[106] Schwarz F., Jennewein M., Bubel M., Holstein J.H., Pohlemann T., Oberringer M. (2013). Soft tissue fibroblasts from well healing and chronic human wounds show different rates of myofibroblasts in vitro. Mol. Biol. Rep..

[107] Kibet M., Abebayehu D. (2025). Crosstalk between T cells and fibroblasts in biomaterial-mediated fibrosis. Matrix Biol. Plus.

[108] Roozendaal R., Mebius R.E. (2011). Stromal cell-immune cell interactions. Annu. Rev. Immunol..

[109] Turley S.J., Cremasco V., Astarita J.L. (2015). Immunological hallmarks of stromal cells in the tumour microenvironment. Nat. Rev. Immunol..

[110] Kraman M., Bambrough P.J., Arnold J.N., Roberts E.W., Magiera L., Jones J.O., Gopinathan A., Tuveson D.A., Fearon D.T. (2010). Suppression of antitumor immunity by stromal cells expressing fibroblast activation protein-α. Science.

[111] Denton A.E., Roberts E.W., Linterman M.A., Fearon D.T. (2014). Fibroblastic reticular cells of the lymph node are required for retention of resting but not activated CD8^+^ T cells. Proc. Natl. Acad. Sci. USA.

[112] Link A., Vogt T.K., Favre S., Britschgi M.R., Acha-Orbea H., Hinz B., Cyster J.G., Luther S.A. (2007). Fibroblastic reticular cells in lymph nodes regulate the homeostasis of naive T cells. Nat. Immunol..

[113] Meng X.M., Nikolic-Paterson D.J., Lan H.Y. (2014). Inflammatory processes in renal fibrosis. Nat. Rev. Nephrol..

[114] Lowes M.A., Suarez-Farinas M., Krueger J.G. (2014). Immunology of psoriasis. Annu. Rev. Immunol..

[115] Wynn T.A., Vannella K.M. (2016). Macrophages in tissue repair, regeneration, and fibrosis. Immunity.

[116] Lech M., Anders H.J. (2013). Macrophages and fibrosis: How resident and infiltrating mononuclear phagocytes orchestrate all phases of tissue injury and repair. BBA Mol. Basis Dis..

[117] Garantziotis S. (2021). Myofibroblast-macrophage interactions turn sour in fibrotic lungs. Am. J. Resp. Cell Mol..

[118] Comito G., Giannoni E., Segura C.P., Barcellos-de-Souza P., Raspollini M.R., Baroni G., Lanciotti M., Serni S., Chiarugi P. (2014). Cancer-associated fibroblasts and M2-polarized macrophages synergize during prostate carcinoma progression. Oncogene.

[119] Schäfer M., Werner S. (2008). Cancer as an overhealing wound: An old hypothesis revisited. Nat. Rev. Mol. Cell Biol..

[120] Kolaczkowska E., Kubes P. (2013). Neutrophil recruitment and function in health and inflammation. Nat. Rev. Immunol..

[121] Obayashi Y., Yamadori I., Fujita J., Yoshinouchi T., Ueda N., Takahara J. (1997). The role of neutrophils in the pathogenesis of idiopathic pulmonary fibrosis. Chest.

[122] Gregory A.D., Kliment C.R., Metz H.E., Kim K.H., Kargl J., Agostini B.A., Crum L.T., Oczypok E.A., Oury T.A., Houghton A.M. (2015). Neutrophil elastase promotes myofibroblast differentiation in lung fibrosis. J. Leukoc. Biol..

[123] Chrysanthopoulou A., Mitroulis I., Apostolidou E., Arelaki S., Mikroulis D., Konstantinidis T., Sivridis E., Koffa M., Giatromanolaki A., Boumpas D.T. (2014). Neutrophil extracellular traps promote differentiation and function of fibroblasts. J. Pathol..

[124] Komi D.E.A., Redegeld F.A. (2020). Role of mast cells in shaping the tumor microenvironment. Clin. Rev. Allergy Immunol..

[125] Krystel-Whittemore M., Dileepan K.N., Wood J.G. (2015). Mast cell: A multi-functional master cell. Front. Immunol..

[126] Wygrecka M., Dahal B.K., Kosanovic D., Petersen F., Taborski B., von Gerlach S., Didiasova M., Zakrzewicz D., Preissner K.T., Schermuly R.T. (2013). Mast cells and fibroblasts work in concert to aggravate pulmonary fibrosis: Role of transmembrane SCF and the PAR-2/PKC-α/Raf-1/p44/42 signaling pathway. Am. J. Pathol..

[127] Garbuzenko E., Nagler A., Pickholtz D., Gillery P., Reich R., Maquart F.X., Levi-Schaffer F. (2002). Human mast cells stimulate fibroblast proliferation, collagen synthesis and lattice contraction: A direct role for mast cells in skin fibrosis. Clin. Exp. Allergy.

[128] Abe M., Kurosawa M., Ishikawa O., Miyachi Y., Kido H. (1998). Mast cell tryptase stimulates both human dermal fibroblast proliferation and type I collagen production. Clin. Exp. Allergy.

[129] Overed-Sayer C., Rapley L., Mustelin T., Clarke D.L. (2013). Are mast cells instrumental for fibrotic diseases?. Front. Pharmacol..

[130] Saalbach A., Klein C., Sleeman J., Sack U., Kauer F., Gebhardt C., Averbeck M., Anderegg U., Simon J.C. (2007). Dermal fibroblasts induce maturation of dendritic cells. J. Immunol..

[131] Marsland B.J., Bättig P., Bauer M., Ruedl C., Lässing U., Beerli R.R., Dietmeier K., Ivanova L., Pfister T., Vogt L. (2005). CCL19 and CCL21 induce a potent proinflammatory differentiation program in licensed dendritic cells. Immunity.

[132] Kamada N., Hisamatsu T., Okamoto S., Sato T., Matsuoka K., Arai K., Nakai T., Hasegawa A., Inoue N., Watanabe N. (2005). Abnormally differentiated subsets of intestinal macrophage play a key role in Th1-dominant chronic colitis through excess production of IL-12 and IL-23 in response to bacteria. J. Immunol..

[133] Fletcher A.L., Lukacs-Kornek V., Reynoso E.D., Pinner S.E., Bellemare-Pelletier A., Curry M.S., Collier A.R., Boyd R.L., Turley S.J. (2010). Lymph node fibroblastic reticular cells directly present peripheral tissue antigen under steady-state and inflammatory conditions. J. Exp. Med..

[134] Roma S., Carpen L., Raveane A., Bertolini F. (2021). The dual role of innate lymphoid and natural killer cells in cancer. From phenotype to single-cell transcriptomics, functions and clinical uses. Cancers.

[135] Spaggiari G.M., Capobianco A., Abdelrazik H., Becchetti F., Mingari M.C., Moretta L. (2008). Mesenchymal stem cells inhibit natural killer-cell proliferation, cytotoxicity, and cytokine production: Role of indoleamine 2,3-dioxygenase and prostaglandin E2. Blood.

[136] Ziani L., Ben Safta-Saadoun T., Gourbeix J., Cavalcanti A., Robert C., Favre G., Chouaib S., Thiery J. (2017). Melanoma-associated fibroblasts decrease tumor cell susceptibility to nk cell-mediated killing through matrix-metalloproteinases secretion. Oncotarget.

[137] Melhem A., Muhanna N., Bishara A., Alvarez C.E., Ilan Y., Bishara T., Horani A., Nassar M., Friedman S.L., Safadi R. (2006). Anti-fibrotic activity of NK cells in experimental liver injury through killing of activated HSC. J. Hepatol..

[138] Paolini R., Bernardini G., Molfetta R., Santoni A. (2015). Nk cells and interferons. Cytokine Growth Factor Rev..

[139] Salimi M., Barlow J.L., Saunders S.P., Xue L.Z., Gutowska-Owsiak D., Wang X.W., Huang L.C., Johnson D., Scanlon S.T., McKenzie A.N.J. (2013). A role for IL-25 and IL-33-driven type-2 innate lymphoid cells in atopic dermatitis. J. Exp. Med..

[140] Kim B.S., Siracusa M.C., Saenz S.A., Noti M., Monticelli L.A., Sonnenberg G.F., Hepworth M.R., Van Voorhees A.S., Comeau M.R., Artis D. (2013). Tslp elicits IL-33-independent innate lymphoid cell responses to promote skin inflammation. Sci. Transl. Med..

[141] Vivier E., Artis D., Colonna M., Diefenbach A., Di Santo J.P., Eberl G., Koyasu S., Locksley R.M., McKenzie A.N.J., Mebius R.E. (2018). Innate lymphoid cells: 10 years on. Cell.

[142] Hams E., Armstrong M.E., Barlow J.L., Saunders S.P., Schwartz C., Cooke G., Fahy R.J., Crotty T.B., Hirani N., Flynn R.J. (2014). IL-25 and type 2 innate lymphoid cells induce pulmonary fibrosis. Proc. Natl. Acad. Sci. USA.

[143] Goto Y., Obata T., Kunisawa J., Sato S., Ivanov I.I., Lamichhane A., Takeyama N., Kamioka M., Sakamoto M., Matsuki T. (2014). Innate lymphoid cells regulate intestinal epithelial cell glycosylation. Science.

[144] Monticelli L.A., Sonnenberg G.F., Abt M.C., Alenghat T., Ziegler C.G.K., Doering T.A., Angelosanto J.M., Laidlaw B.J., Yang C.Y., Sathaliyawala T. (2011). Innate lymphoid cells promote lung-tissue homeostasis after infection with influenza virus. Nat. Immunol..

[145] Mchedlidze T., Waldner M., Zopf S., Walker J., Rankin A.L., Schuchmann M., Voehringer D., McKenzie A.N.J., Neurath M.F., Pflanz S. (2013). Interleukin-33-dependent innate lymphoid cells mediate hepatic fibrosis. Immunity.

[146] Dahlgren M.W., Jones S.W., Cautivo K.M., Dubinin A., Ortiz-Carpena J.F., Farhat S., Yu K.S., Lee K., Wang C.Q., Molofsky A.V. (2019). Adventitial stromal cells define group 2 innate lymphoid cell tissue niches. Immunity.

[147] François A., Chatelus E., Wachsmann D., Sibilia J., Bahram S., Alsaleh G., Gottenberg J.E. (2013). B lymphocytes and B-cell activating factor promote collagen and profibrotic markers expression by dermal fibroblasts in systemic sclerosis. Arthritis Res. Ther..

[148] Matsushita T., Hasegawa M., Yanaba K., Kodera M., Takehara K., Sato S. (2006). Elevated serum baff levels in patients with systemic sclerosis—Enhanced BAFF signaling in systemic sclerosis B lymphocytes. Arthritis Rheum..

[149] Barone F., Gardner D.H., Nayar S., Steinthal N., Buckley C.D., Luther S.A. (2016). Stromal fibroblasts in tertiary lymphoid structures: A novel target in chronic inflammation. Front. Immunol..

[150] Cremasco V., Woodruff M.C., Onder L., Cupovic J., Nieves-Bonilla J.M., Schildberg F.A., Chang J., Cremasco F., Harvey C.J., Wucherpfennig K. (2014). B cell homeostasis and follicle confines are governed by fibroblastic reticular cells. Nat. Immunol..

[151] Kragstrup T.W., Otkjaer K., Hohn C., Jorgensen A., Hokland M., Iversen L., Deleuran B. (2008). The expression of IL-20 and IL-24 and their shared receptors are increased in rheumatoid arthritis and spondyloarthropathy. Cytokine.

[152] Philippeos C., Telerman S.B., Oules B., Pisco A.O., Shaw T.J., Elgueta R., Lombardi G., Driskell R.R., Soldin M., Lynch M.D. (2018). Spatial and single-cell transcriptional profiling identifies functionally distinct human dermal fibroblast subpopulations. J. Investig. Dermatol..

[153] Shi Z., Liu Z., Wei Y., Zhang R., Deng Y., Li D. (2024). The role of dermal fibroblasts in autoimmune skin diseases. Front. Immunol..

[154] Zang R., Xu C.C., Fan Z., Wang Q.N., Guo Z.J., Liu L., Cui B.N., Huang Y.Y., Yang J. (2025). The role of fibroblasts in chronic inflammatory and proliferative skin diseases. Exp. Dermatol..

[155] Albanesi C., Madonna S., Gisondi P., Girolomoni G. (2018). The interplay between keratinocytes and immune cells in the pathogenesis of psoriasis. Front. Immunol..

[156] Chen X.Y., Wu Y.T., Jia S.J., Zhao M. (2024). Fibroblast: A novel target for autoimmune and inflammatory skin diseases therapeutics. Clin. Rev. Allerg. Immunol..

[157] Angiolilli C., Leijten E.F.A., Bekker C.P.J., Eeftink E., Giovannone B., Nordkamp M.O., van der Wal M., Thijs J.L., Vastert S.J., van Wijk F. (2022). ZFP_3_6 family members regulate the proinflammatory features of psoriatic dermal fibroblasts. J. Investig. Dermatol..

[158] Kim J., Lee J., Kim H.J., Kameyama N., Nazarian R., Der E., Cohen S., Guttman-Yassky E., Putterman C., Krueger J.G. (2021). Single-cell transcriptomics applied to emigrating cells from psoriasis elucidate pathogenic versus regulatory immune cell subsets. J. Allergy Clin. Immunol..

[159] Detmar M., Brown L.F., Claffey K.P., Yeo K.T., Kocher O., Jackman R.W., Berse B., Dvorak H.F. (1994). Overexpression of vascular permeability factor/vascular endothelial growth factor and its receptors in psoriasis. J. Exp. Med..

[160] Langan S.M., Irvine A.D., Weidinger S. (2020). Atopic dermatitis. Lancet.

[161] Weidinger S., Beck L.A., Bieber T., Kabashima K., Irvine A.D. (2018). Atopic dermatitis. Nat. Rev. Dis. Primers.

[162] Brandt E.B., Sivaprasad U. (2011). Th2 cytokines and atopic dermatitis. J. Clin. Cell Immunol..

[163] Denton C.P., Khanna D. (2017). Systemic sclerosis. Lancet.

[164] Varga J., Abraham D. (2007). Systemic sclerosis: A prototypic multisystem fibrotic disorder. J. Clin. Investig..

[165] Assassi S., Swindell W.R., Wu M., Tan F.D., Khanna D., Furst D.E., Tashkin D.P., Jahan-Tigh R.R., Mayes M.D., Gudjonsson J.E. (2015). Dissecting the heterogeneity of skin gene expression patterns in systemic sclerosis. Arthritis Rheumatol..

[166] Tabib T., Huang M., Morse N., Papazoglou A., Behera R., Jia M., Bulik M., Monier D.E., Benos P.V., Chen W. (2021). Myofibroblast transcriptome indicates SFRP2^hi^ fibroblast progenitors in systemic sclerosis skin. Nat. Commun..

[167] Fonacier L., Bernstein D.I., Pacheco K., Holness D.L., Blessing-Moore J., Khan D., Lang D., Nicklas R., Oppenheimer J., Portnoy J. (2015). Contact dermatitis: A practice parameter—update 2015. J. Allergy Clin. Immunol. Pract..

[168] Martin S.F. (2012). Contact dermatitis: From pathomechanisms to immunotoxicology. Exp. Dermatol..

[169] Lee H.Y., Stieger M., Yawalkar N., Kakeda M. (2013). Cytokines and chemokines in irritant contact dermatitis. Mediat. Inflamm..

[170] Kaplan D.H., Igyarto B.Z., Gaspari A.A. (2012). Early immune events in the induction of allergic contact dermatitis. Nat. Rev. Immunol..

[171] Eming S.A., Martin P., Tomic-Canic M. (2014). Wound repair and regeneration: Mechanisms, signaling, and translation. Sci. Transl. Med..

[172] Moretti L., Stalfort J., Barker T.H., Abebayehu D. (2022). The interplay of fibroblasts, the extracellular matrix, and inflammation in scar formation. J. Biol. Chem..

[173] Demidova-Rice T.N., Hamblin M.R., Herman I.M. (2012). Acute and impaired wound healing: Pathophysiology and current methods for drug delivery, part 2: Role of growth factors in normal and pathological wound healing: Therapeutic potential and methods of delivery. Adv. Skin Wound Care.

[174] Ogawa R. (2017). Keloid and hypertrophic scars are the result of chronic inflammation in the reticular dermis. Int. J. Mol. Sci..

[175] Bran G.M., Goessler U.R., Hormann K., Riedel F., Sadick H. (2009). Keloids: Current concepts of pathogenesis (review). Int. J. Mol. Med..

[176] Munavalli G.S., Smith S., Maslowski J.M., Weiss R.A. (2013). Successful treatment of depressed, distensible acne scars using autologous fibroblasts: A multi-site, prospective, double blind, placebo-controlled clinical trial. Dermatol. Surg..

[177] Weiss R.A., Weiss M.A., Beasley K.L., Munavalli G. (2007). Autologous cultured fibroblast injection for facial contour deformities: A prospective, placebo-controlled, phase III clinical trial. Dermatol. Surg..

[178] Marston W.A., Hanft J., Norwood P., Pollak R., Study D.D.F.U. (2003). The efficacy and safety of dermagraft in improving the healing of chronic diabetic foot ulcers—Results of a prospective randomized trial. Diabetes Care.

[179] Veves A., Falanga V., Armstrong D.G., Sabolinski M.L., Apligraf Diabetic Foot Ulcer Study (2001). Graftskin, a human skin equivalent, is effective in the management of noninfected neuropathic diabetic foot ulcers—A prospective randomized multicenter clinical trial. Diabetes Care.

[180] Butler D.L., Juncosa-Melvin N., Boivin G.P., Galloway M.T., Shearn J.T., Gooch C., Awad H. (2008). Functional tissue engineering for tendon repair: A multidisciplinary strategy using mesenchymal stem cells, bioscaffolds, and mechanical stimulation. J. Orthop. Res..

[181] Nesic D., Whiteside R., Brittberg M., Wendt D., Martin I., Mainil-Varlet P. (2006). Cartilage tissue engineering for degenerative joint disease. Adv. Drug Deliv. Rev..

[182] Blauvelt A., Chiricozzi A. (2018). The immunologic role of IL-17 in psoriasis and psoriatic arthritis pathogenesis. Clin. Rev. Allergy Immunol..

[183] Wollenberg A., Beck L.A., Blauvelt A., Simpson E.L., Chen Z., Chen Q., Shumel B., Khokhar F.A., Hultsch T., Rizova E. (2020). Laboratory safety of dupilumab in moderate-to-severe atopic dermatitis: Results from three phase III trials (LIBERTY AD SOLO 1, LIBERTY AD SOLO 2, LIBERTY AD CHRONOS). Br. J. Dermatol..

[184] Schwartz D.M., Bonelli M., Gadina M., O’Shea J.J. (2016). Type I/II cytokines, JAKs, and new strategies for treating autoimmune diseases. Nat. Rev. Rheumatol..

[185] Amrute J.M., Luo X., Penna V., Yang S., Yamawaki T., Hayat S., Bredemeyer A., Jung I.H., Kadyrov F.F., Heo G.S. (2024). Targeting immune-fibroblast cell communication in heart failure. Nature.

[186] Sharpe A.H., Pauken K.E. (2018). The diverse functions of the PD1 inhibitory pathway. Nat. Rev. Immunol..

[187] Richeldi L., du Bois R.M., Raghu G., Azuma A., Brown K.K., Costabel U., Cottin V., Flaherty K.R., Hansell D.M., Inoue Y. (2014). Efficacy and safety of nintedanib in idiopathic pulmonary fibrosis. N. Engl. J. Med..

[188] Justice J.N., Nambiar A.M., Tchkonia T., LeBrasseur N.K., Pascual R., Hashmi S.K., Prata L., Masternak M.M., Kritchevsky S.B., Musi N. (2019). Senolytics in idiopathic pulmonary fibrosis: Results from a first-in-human, open-label, pilot study. eBioMedicine.

[189] Gill S.E., Parks W.C. (2008). Metalloproteinases and their inhibitors: Regulators of wound healing. Int. J. Biochem. Cell Biol..

[190] Trackman P.C. (2016). Lysyl oxidase isoforms and potential therapeutic opportunities for fibrosis and cancer. Expert. Opin. Ther. Targets.

